# Defining Minimum Essential Factors to Derive Highly Pure Human Endothelial Cells from iPS/ES Cells in an Animal Substance-Free System

**DOI:** 10.1038/srep09718

**Published:** 2015-04-13

**Authors:** Yu-Ting Wu, I.-Shing Yu, Kuen-Jer Tsai, Chien-Yu Shih, Shiaw-Min Hwang, Ih-Jen Su, Po-Min Chiang

**Affiliations:** 1 Institute of Clinical Medicine, National Cheng Kung University, Tainan, Taiwan, ROC; 2 Laboratory Animal Center, College of Medicine, National Taiwan University, Taipei, Taiwan, ROC; 3 Food Industry Research and Development Institute, Hsinchu, Taiwan, ROC; 4 Division of Infectious Diseases, National Health Research Institutes, Tainan, Taiwan, ROC

## Abstract

It is desirable to obtain unlimited supplies of endothelial cells for research and therapeutics. However, current methods of deriving endothelial cells from humans suffer from issues, such as limited supplies, contamination from animal substances, and lengthy/complicated procedures. In this article we developed a way to differentiate human iPS and ES cells to highly pure endothelial cells in 5 days. The chemically defined system is robust, easy to perform, and free of animal substances. Using the system, we verified that combined TGFβ and canonical Wnt agonists are essential and sufficient for iPS/ES cell-to-mesoderm transition. Besides, VEGF-KDR signaling alone is required for endothelial formation at high density while supplementation with FGF allows for colonial endothelial differentiation. Finally, anti-adsorptive agents could enrich the endothelial output by allowing selective attachment of the endothelial precursors. The system was validated to work on multiple iPS/ES cells lines to produce endothelial cells capable of forming capillary-like structures *in vitro* and integrating into host vasculature *in vivo*. In sum, the simple yet robust differentiation system permits the unlimited supply of human endothelial cells. The defined and animal substance-free nature of the system is compatible with clinical applications and characterization of endothelial differentiation in an unbiased manner.

Endothelial cells, an essential component of blood vessels, are required for blood supply in all organs. Lining on the inner surfaces of vessels, they regulate the traffic of nutrients and cells between blood and tissues^1^,^2^. In addition, by signaling to the smooth muscles or pericytes on the vascular walls, they regulate vessel tone and, thus, the oxygen and nutrient supply. Pathologically, dysfunctional endothelium is involved in diseases such as atherosclerosis and hypertension^3^. Therefore, it is important to obtain functional human endothelial cells to understand the cells. Besides, generating high-purity endothelial cells in large quantity is useful in terms of clinical applications for ischemia or engineered organs^4^,^5^.

The derivation of isogenic endothelial cells directly from humans is currently limited by the inability to maintain and amplify those cells for long time *ex vivo*^6^. This limitation can be solved theoretically by differentiating induced pluripotent (iPS) cells *in vitro*^7^, because iPS cells could be derived from somatic cells in an integration-free manner^8^ and be amplified unlimitedly *in vitro* in a chemically defined system^9^,^10^. However, to differentiate iPS cells or embryonic stem (ES) cells into endothelial cells, current methods suffer from issues of low purity and carryover from animal substances. The low purity of target endothelial cells during differentiation requires sorting or other ways to isolate them, which complicates the process to scale the system up. The unavoidable use of animal-sourced ingredients leads to issues of infectious contamination^11^ and variation in efficiency due to batch-to-batch inconsistency^12^ in the differentiation process. Thus, a simple and scalable method to differentiate human iPS/ES cells into endothelial cells is much needed.

The formation of endothelial cells from embryonic stem cells goes through an intermediate stage of mesoderm^13^. Based on the signals known for gastrulation and vasculogenesis *in vivo*, FGFs, Wnts, TGFs, and BMPs are commonly used to allow the formation of KDR+ hemangioblasts^14^,^15^,^16^,^17^, the endothelial precursor in mesoderm^18^. FGFs, VEGF, BMPs, and inhibitors of TGF signaling pathways are used to differentiate hemangioblasts into mature endothelial cells^7^,^19^,^20^. In addition to the specific molecules, the differentiation system often includes various undefined substances such as Matrigel, Sera, or proprietary culture media. These substances usually contain signaling molecules that affect differentiation^21^,^22^, which in turn prevents us from defining the minimal yet essential set of exogenous factors that drive endothelial formation. Further, the undefined system could contain signals that drives the formation of lineages other than endothelium^7^, which reduces the purity and yield of the desirable endothelial cells.

Here we developed a simple and animal substance-free differentiation system to acquire high-purity endothelial cells from human iPS and ES cells in 5 days. The system is simple so that only one split is required during differentiation. Its formulation is completely defined and free of animal substances. The endothelial identity, validated by both *in vitro* and *in vivo* assays, could be obtained in high purity in a short differentiation time and at a colonial density. The simplicity and scalable potential are compatible with clinical applications, and its defined and low-density nature will enable easier characterization of endothelial differentiation mechanistically in the future.

## Results

### The treatment of a glycogen synthase kinase inhibitor and a TGFβ agonist triggered mesoderm formation

Both TGFβ^23^ and Wnt^24^,^25^ signaling pathways are known to be critical for the mesodermal transition during gastrulation. Also, the 2 pathways are required for epiblast-to-mesoderm transition for differentiation of murine cells *in vitro*^26^. Accordingly, we tested if a combination of the 2 agonists could differentiate the iPS cells into mesoderm, the precursor of endothelial cells.

**Figure 1:**
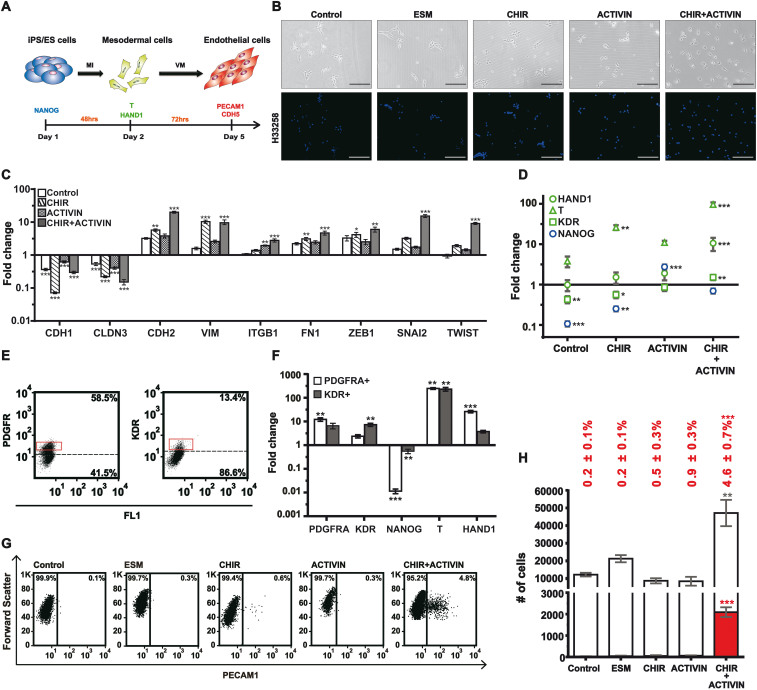
The requirement of TGFβ and Wnt agonists for the formation of mesoderm. (A) Timeframe involved in the stepwise differentiation from iPS/ES cells into endothelial cells. The representative markers of each cell type were labeled beneath them. MI: mesoderm inducer; VM: vasculogenic mixture. (B) Morphologies (upper panels: phase-contrast images; lower panels: nuclear staining) of the iPS cells induced under various conditions. Conditions were: Control (basal medium plus 10 µM of Y27632); ESM (the TeSR-E8 plus 10 µM of Y-27632); CHIR (Control plus 3 µM of CHIR99021); ACTIVIN (Control plus 2 ng/ml of Activin A). iPS cells (10,000) were seeded per well of a 12-well plate in the conditions and assayed 48 hours later. Scale bar: 200 µm. Hoechst 33258 served as the nuclear stain. (C) and (D) The cells were induced as in B and harvested after 48 hours. The mRNA levels of CDH1, CLDN3, CDH2, VIM, ITGB1, FN1, ZEB1, SNAI2, TWIST1 (C), HAND1, T, KDR, and NANOG (D) in each group relative to the iPS cells (ESM) were assayed. GAPDH served as a loading control. (E) Fluorescence-activated cell sorting for PDGFR+ (left) and KDR+ (right) cells. iPS cells were induced as the CHIR+ACTIVIN group in B and stained for sorting 48 hours later. (F) The cells in the red boxes of E were assayed for the mRNA levels of PDGFRA, KDR, NANOG, T, and HAND1 relative to the iPS cells. GAPDH served as a loading control. (G) Representative flow cytometry analyses for PECAM1+ cells on day 5 when the mesoderm was induced under the conditions in B. The iPS cells were induced as in B for 48 hours and the media were replaced with the basal medium plus 20 ng/ml of mVEGF-A for additional 72 hours before flow cytometry. (H) Quantification (mean ± s.e.m., n = 3) of G for the number of PECAM1+ cells (red columns) and the number of total cells (red plus white) on day 5. Percentages (mean ± s.e.m., n = 3) above each bar represent the ratios of PECAM1+ cell number relative to the total cell number. * denotes a significant difference compared with the control.

Morphologically, when the dissociated iPS cells were seeded in BM (Fig. 1B; Control) or BM plus either a TGFβ agonist, Activin A (Fig. 1B; ACTIVIN), or a canonical-Wnt agonist, CHIR99021^27^ (Fig. 1B; CHIR), the cells remained attached to each other, similar to those cultured in the ES medium (Fig. 1B; ESM). In contrast, the presence of both Activin A and CHIR99021 triggered mutual detachment of the cells (Fig. 1B; ACTIVIN+CHIR). To assay for mesenchymal transition at mRNA expression level, the cells of each group were harvested to assay for a panel of epithelial (CDH1 and CLDN3)^28^ and mesenchymal (CDH2, VIM, ITGB1, FN1, ZEB, SNAI2, and TWIST1)^29^,^30^ markers by reverse-transcriptase quantitative polymerase chain reaction (RT-qPCR). Compared with the iPS cells, the removal of ES medium lead to significant reduction of the epithelial markers (Fig. 1C; CDH1 and CLDN3; all 4 groups). The inclusion of Activin A (Fig. 1C; ITGB1; ACTIVIN) or CHIR99021 (Fig. 1C; CDH2, VIM, FN1, ZEB1; CHIR) alone caused increased expression of some mesenchymal markers. However, only the combination of Activin A and CHIR99021 resulted in significant elevation of SNAI2 and TWIST1 (Fig. 1C; SNAI2 and TWIST1; CHIR+ACTIVIN) besides all the other mesenchymal markers. Together, the data showed that the inclusion of both agonists triggered significant mesenchymal transition^31^.

The induced cells were further assayed for the expression of NANOG, HAND1, T, and KDR to validate mesoderm formation. In contrast to the control cells (Fig. 1D; Control), or cells induced with Activin A (Fig. 1D; ACTIVIN) or CHIR99021 (Fig. 1D; CHIR) alone, those induced with BM plus both agonists exhibited elevated expression of all three mesodermal markers, T, HAND1 and KDR (Fig. 1D; iPS versus ACTIVIN+CHIR; one-way ANOVA Tukey's *P* <0.05 for all 3 genes). In addition to the induction of mesodermal markers in the pooled cells, the combined induction with both agonists also triggered the formation of PDGFRA-expressing and a minor population of KDR+ cells (Fig. 1E; left and right, respectively) 48 hours later. Relative to the iPS cells, the sorted PDGFRA+ and KDR+ cells showed reduced mRNA levels of NANOG (Fig. 1F; NANOG; PDGFR+ and KDR+) and increased expression of mesodermal markers, T (Fig. 1F; PDGFRA+ and KDR+) and HAND1 (Fig 1F; PDGFRA+). In sum, the induced expression of multiple mesodermal markers^32^ in terms of both mRNA transcription and surface-marker expression demonstrated that the combined treatment of Activin A and CHIR99021 drove iPS cells to mesoderm.

To confirm that Activin A and CHIR99021 together could truly induce endothelial precursors in the converted mesoderm, we induced the iPS cells with the identical conditions above for 48 hours and replaced the media with BM plus murine VEGF-A (mVEGF-A) for additional 72 hours. The resultant cells were assayed for the presence of endothelial cells by flow cytometry for the positivity of PECAM1, an endothelial marker (Fig. 1G). A significant amount of endothelial formation was only observed when both Activin A and CHIR99021 were present during the first 48 hours of differentiation (Fig. 1H; numbers of endothelial cells; ACTIVIN+CHIR versus the other 4 groups; 2090 ± 227 versus <70; one-way ANOVA Turky's *P*<0.01).

In summary, the combination of Activin A and CHIR99021 were required for the formation of mesoderm as well as endothelial precursors in our chemically defined system. The successful iPS cells-to-mesoderm transition were demonstrated by the increased cell dispersion, the elevated expression of mesenchymal/mesodermal markers, and the potential to form endothelial cells. Further, the chemically defined nature of the system also supported the sufficiency of the 2 agonists in converting iPS cells into mesoderm.

### Anti-adsorptives dramatically improved the purity of endothelial cells by selectively allowing the attachment of their precursors.

**Figure 2:**
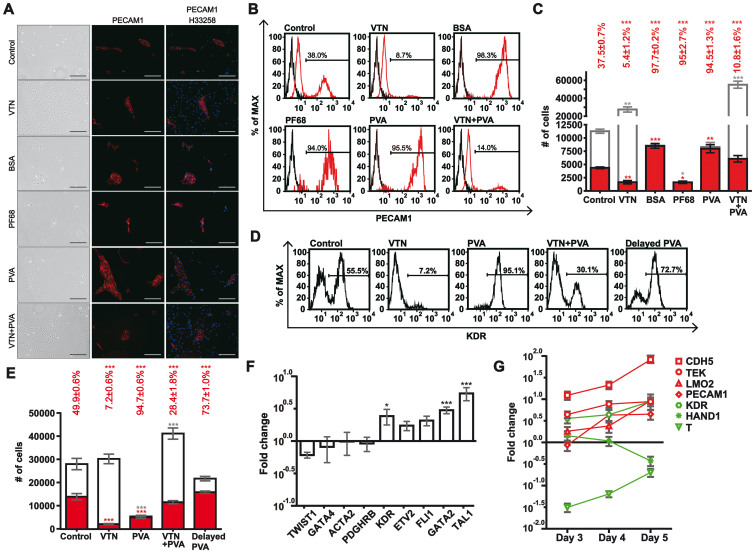
The effect of anti-adsorptives on the yield and purity of the differentiated endothelial cells. (A) Phase-contrast images (left) and staining for PECAM1 immunopositivity (middle and right, red) of the cells on day 5. iPS cells were induced with MI for 48 hours and replated with various factors for additional 72 hours. The factors were: Control: basal medium plus mVEGF-A; VTN: Control with VTN; BSA: Control plus BSA; PF68: Control plus PF68; PVA: Control plus PVA. Scale bars: 200 µm. The H33258 served as the nuclear counterstain in the right panels. (B) Representative flow cytometry of A for PECAM1+ cells on day 5. Red and black curves represent staining with PE-labeled anti-PECAM1 antibody and unstained control, respectively. (C) Quantification of B for the number of PECAM1+ cells (red) and the number of total cells (red plus white) on day 5. Percentages above each bar represent the PECAM1+ cell number relative to the total cell number. (D) Representative flow cytometry analyses for KDR+ cells on day 3. Mesodermal cells were replated with various factors for additional 24 hours. The factors were: Control: basal medium plus mVEGF-A; VTN: Control with VTN; PVA: Control plus PVA; delayed PVA: Control with PVA added 6 hours after seeding. (E) Quantification of D for the number of KDR+ cells (red) and the number of total cells (red plus white) on day 3. Percentages above each bar represent KDR+ cell number relative to the total cell number. (F) Gene expression profiling of the PVA-enriched adherent cells on day 3. Mesodermal cells were replated in VM or VM minus PVA (control) for 24 hours. The adherent cells were harvested to compare for the expression levels. GAPDH served as a loading control. (G) Gene expression profiling of the cells on days 3–5 relative to the day-2 mesoderm. Mesodermal cells were replated in VM for 3 days. The adherent cells were harvested daily from day 2 to 5 for the expression levels. GAPDH served as a loading control. *A significant difference when compared with the control by Tukey's post-ANOVA test. All values represent mean ± s.e.m., n = 3.

The yield (2089 ± 227 of endothelial cells per 10,000 iPS-cell input) and the purity (4.6 ± 0.7%) of the endothelial cells by the process above were low. To improve the efficiency, we tried to remove the VTN from the culture by replating the mesodermal cells onto uncoated culture dishes on day 2. The removal of VTN from culture improved the purity of endothelial cells 72 hours after replating (Figs. 2A–C; percentage of PECAM1+ cells; Control versus VTN; 37.5 ± 0.7% versus 5.4 ± 1.2%; one-way ANOVA Tukey's *P*<0.001). The improvement suggested that selective attachment would be a way to enhance the purity of endothelial cells, and the endothelial cells or their precursors were able to attach to the plastics under more stringent conditions. To make the culture system more stringent against attachment, we seeded the dissociated mesoderm on day 2 to media containing the following anti-adsorption agents: Pluronic F-68 (PF68)^33^, Poly(vinyl alcohol) (PVA)^34^, or bovine serum albumin (BSA)^35^. Each of them dramatically improved the purity of the endothelial cells 72 hours after replating (Figs. 2A–C; percentage of PECAM1+ cells; PF68, PVA, or BSA versus Control; 95.0 ± 2.7%, 94.5 ± 1.3%, 97,7 ± 0.2% versus 37.5 ± 0.7%; one-way ANOVA Tukey's *P*< 0.001). However, the yield of PECAM1+ endothelial cells with PF68 was significantly lower than those with BSA and PVA (Figs. 2C; number of PECAM1+ cells; PF68 vs PVA and BSA; 1478 ± 226 versus 7832 ± 774 and 8359 ± 434; one-way ANOVA Tukey's *P*< 0.05). We chose to use PVA because it is synthetic, which avoided concerns of undefined carryovers during BSA purification and issues of infectious contamination.

To refine the timing PVA exerted its purifying effect, we replated day-2 mesodermal cells in combinations of VTN and PVA and then characterized the purity/number of adherent KDR+ cells 24 hours later. The coating of VTN significantly reduced the purity of KDR+ endothelial precursors after replating (Figs. 2D–E; percentage of KDR+ cells; VTN versus Control; 7.2 ± 0.6% versus 49.9 ± 0.6%; one-way ANOVA Tukey's *P*<0.001), consistent with the low purity of PECAM1+ endothelial cells 72 hours after replating. The presence of PVA dramatically improved the purity of adherent KDR+ cells within 24 hours after replating (Figs. 2D–E; percentage of KDR+ cells; PVA versus Control; 94.7 ± 0.6% Vs 49.9 ± 0.6%; one-way ANOVA Tukey's *P*<0.001) at the expense of reduced KDR+ cell numbers (Figs. 2D–E; number of KDR+ cells; PVA versus Control; 5079 ± 436 versus 13989 ± 1366; one-way ANOVA Tukey's *P*<0.001). Further, PVA also enhanced the purity of KDR+ cells on VTN-coated plates (Figs. 2D–E; percentage of KDR+ cells; PVA+VTN versus VTN; 28.4 ± 1.8% versus 7.2 ± 0.6%; one-way ANOVA Tukey's *P*<0.001). Further, the purifying effect was reduced if the PVA was added 6 hours after replating (Figs. 2D–E; percentage of KDR+ cells; delayed PVA vs PVA; 73.7 ± 1.0% versus 94.7 ± 0.6%; one-way ANOVA Tukey's *P*<0.001).

In addition to KDR, we measured the expression levels of several mesodermal, hemangioblastic, and endothelial markers in the PVA-enriched adherent cells on day 3. By RT-qPCR assays, the expression levels of hemangioblastic markers KDR, GATA2 and TAL1 were significantly higher with the inclusion of PVA (Fig. 2F; PVA versus Control; one-way ANOVA Tukey's *P*< 0.05). The trend of enrichment was also observed with makers such as ETV2, and FLI1. In contrast, those markers belonging to early or other mesodermal lineages were either comparably reduced or unchanged (Fig. 2F; PVA versus Control; TWIST1, GATA4, ACTA2, and PDGFRB). The increasing expression levels of endothelial markers, such as PECAM1, CDH5, and LMO2, were also demonstrated by assaying the PVA-enriched adherent cells for 3 consecutive days (Fig. 2G; the cells of days 3–5 versus day-2 mesoderm).

Taken together, the presence of VTN significantly reduced both the quantity and purity of endothelial cells after the stage of mesoderm. Removal of VTN by replating plus the inclusion of PVA helped acquire high-purity endothelial cells. The enrichment occurred early after replating. The purifying effects of PVA were possibly mediated by inhibiting the attachment of cells other than KDR+ cells.

### VEGF-KDR and basic FGF pathways allowed for colonial differentiation from mesoderm to endothelial cells

**Figure 3:**
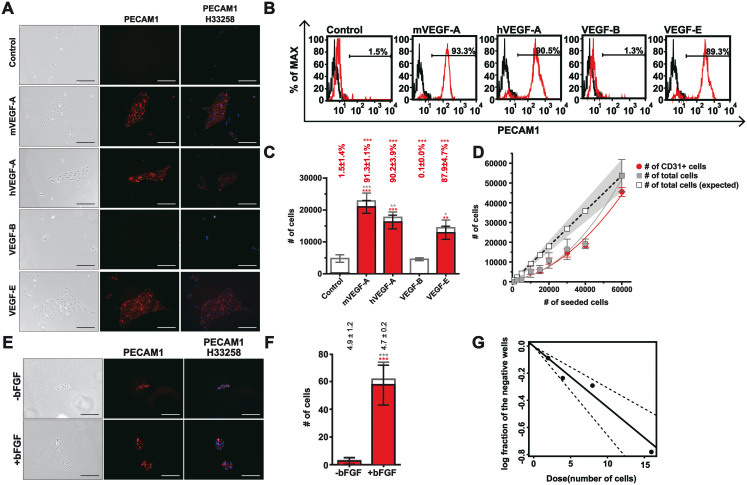
The requirement of VEGF and FGF pathways for mesoderm-to-endothelium transition. (A) Phase-contrast images (left) and immunofluorescence for PECAM1 immunopositivity (middle and right, red) of cells on day 5. Mesodermal cells were replated with various factors for 72 hours. The factors: Control: basal medium plus PVA; mVEGF-A: Control plus mVEGF-A; hVEGF-A: Control plus hVEGF-A; VEGF-B: Control plus VEGF-B; VEGF-E: Control plus VEGF-E. The H33258 served as the nuclear counterstain. (B) Representative flow cytometry of A for PECAM1+ cells on day 5. Red and black curves represent staining with PE-labeled anti-PECAM1 antibody and unstained control, respectively. (C) Quantification of B for the number of PECAM1+ cells (red) and the number of total cells (red plus white). Percentages above each bar represent the ratios of PECAM1+ cell number relative to the total cell number. *Significance when compared with the control by Tukey's post-ANOVA test. (D) The expected (hollow squares) and actual (solid squares: total cells; red circles: PECAM1+ cells) numbers of the induced cells on day 5 under serial seeding densities. Mesodermal cells (1,000 to 60,000) were replated with VM for 72 hours before harvested for assay. The expected cell numbers and the corresponding confidence intervals (gray shade) were based on the 60,000 input density. (E) Phase-contrast images (left) and immunofluorescence staining for PECAM1+ colonies (middle and right) in the absence (−) or presence (+) of basic FGF (bFGF). Mesodermal cells (1,000) were replated in VM with or without bFGF for 72 hours before staining for PECAM1 immunopositivity. H33258 served as the nuclear stain. (F) Quantification of PECAM1+ (red) and total (red plus white) colony numbers in E. The numbers above each column represent the average numbers of cells per colony. *Significance by Student's *t*-test. (G) Limiting dilution assay for the colonial frequency with bFGF. Mesodermal cells were replated in VM plus bFGF in a 96-well plate (2, 4, 8, or 16 cells per well) for 72 hours. Twenty-four wells were seeded for each density and log fractions of the negative wells were plotted against the input. All values represent mean ± s.e.m., n = 3. Scale bars = 200 µm.

The mVEGF-A used to drive endothelial formation was insect cell-derived and known to activate both VEGFR-1^36^ and KDR (VEGFR-2)^37^. To remove all animal substances and to further clarify how VEGF-A drove endothelial differentiation, we substituted the mVEGF-A with several other members of the VEGF family. As expected, *E. coli*-derived human VEGF-A (hVEGF-A) was able to replace mVEGF-A in driving the formation of endothelial cells (Figs. 3A–C; number of PECAM1+ cells; mVEGF-A and hVEGF-A vs Control; 20788 ± 2006 and 16041 ± 2147 versus 43 ± 34; one-way ANOVA Tukey's *P*<0.001). The replacement of insect cell-derive mVEGF-A with E. coli-derived hVEGF-A made our differentiation process completely defined and free of animal substances.

In contrast to human or mouse VEGF-A, VEGF-B failed to drive endothelial formation (Figs. 3A–C; number of PECAM1+ cells; VEGF-B versus mVEGF-A; 7 ± 1 versus 20788 ± 2006; one-way ANOVA Tukey's P<0.001). In contrast, the purity of endothelial formation with *E. coli*-derived VEGF-E was comparable to that of mVEGF-A (Figs. 3A–C; percentage of PECAM1+ cells; VEGF-E versus Control; 87.9 ± 4.7% versus 1.5 ± 1.4%; one-way ANOVA Tukey's *P*<0.001), albeit with a lower yield (Fig. 3C; number of PECAM1+ cells; VEGF-E versus mVEGF-A; 12657 ± 2073 versus 20788 ± 2006; one-way ANOVA Tukey's P<0.05). The restricted endothelium-driving capability by VEGF-E, a KDR selective agonist^38^, but not VEGF-B, an Flt-1 selective agonist^39^, suggested KDR was the receptor mediating endothelial formation in our chemically defined system.

To test the sufficiency of VEGF in driving the mesoderm-to-endothelium transition, we differentiated the day-2 mesoderm into endothelium at various seeding densities. The output of endothelial cells decreased disproportionally when the numbers of input cells decreased (Fig. 3D; actual versus expected cell numbers). The deviation of the actual yield from linearity suggested mutual communications between cells enhanced their survival during mesoderm-to-endothelial transition. Mesodermal cells secret FGF^40^ and FGF signaling plays critical roles in mesoderm survival^41^, hemangioblast expansion^42^, and endothelial culture^43^. Further, human basic FGF (bFGF) was used to drive fibroblast-derived angioblast-like cells to endothelium^44^. Thus, we supplemented bFGF to see if it increased endothelial output at a low seeding density. With the inclusion of bFGF, mesodermal cells, albeit seeded at low density, formed endothelial colonies (Fig. 3E) more efficiently compared with the control (Fig. 3F; number of PECAM1+ colonies; +bFGF versus –bFGF; 57.3 ± 1 versus 2.3 ± 0.9; Student's *t*-test *P*<0.001). In contrast to the low number of colonies, the numbers of cells per colony were similar between the 2 groups (Fig. 3F; number of cells per colony; +bFGF versus –bFGF; 4.7 ± 0.2 versus 4.9 ± 1.2). The colony formation frequency with bFGF supplementation was 1 in 22.3 by limiting dilution assays (Fig. 3G; confidence interval of the frequency: 1 in 15.2 to 32.6)^45^.

In sum, VEGF was sufficient to drive mesodermal-to-endothelium transition at high seeding density. The key receptor responsible for the transition was likely KDR. Further, FGF signaling was another key signal to allow for colonial differentiation of endothelial colonies at ~5% successful rates by both low-density culture and limiting dilution assay.

### *In vitro* and *in vivo* characterizations confirmed the generality of the method and the identity of the differentiated human endothelium

**Figure 4:**
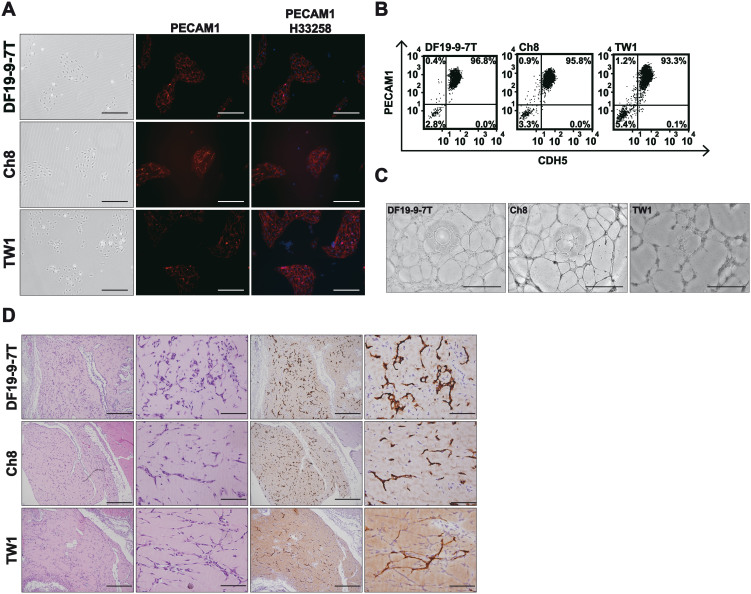
The validation of endothelial identity across multiple iPS/ES cell lines. (A) Phase-contrast images (left panels) and immunofluorescence for PECAM1+ cells (middle and right panels, red) on day 5. Per well of a 12-well plate, iPS (DF19-9-7T) and ES (Ch8, and TW1) cells (20,000) were induced with MI for 48 hours and replated (30,000) in VM for additional 72 hours before staining for PECAM1 immunopositivity. H33258 served as the nuclear stain. Scale bars = 200 µm. (B) Representative flow cytometry for PECAM1+ and CDH5+ cells on day 5. iPS (DF19-9-7T) and ES (Ch8, and TW1) cells were induced as in A and harvested for staining with PE-conjugated anti-PECAM1 and FITC-conjugated anti-CDH5 on day 5. (C) Matrigel capillary assays for cell lines DF19-9-7T (left), Ch8 (middle), and TW1 (right). Per well of a 6-well plate, iPS/ES cells (40,000) were induced with MI for 48 hours and replated (60,000) in VM for additional 72 hours. On day 5, the cells were dissociated and seeded on Matrigel matrix (45,000/well in a 96-well plate) for the formation of capillary like-structures. Scale bars = 1 mm. (D) H&E (left panels) and anti-hPECAM1 immunohistochemical (right panels) staining of the injected Matrigel plugs. Per 10 cm dish, iPS/ES cells (300,000) were induced with MI for 48 hours and replated (900,000) in VM for additional 72 hours. The cells were dissociated and mixed with Matrigel matrix for subcutaneous injection in immunodeficient mice on day 5. The plugs were harvested 7 days after injection. Scale bars (low power) = 1 mm; scale bars (high power) = 200 µm.

To validate the generality of the differentiation system across multiple iPS/ES cell lines, we incorporated two other human ES cell lines, TW1 and Ch8, in addition to the iPS cell line DF19-9-7T used for developing the method. With the identical differentiation method, endothelial cells could form within 5 days with all three cell lines (Table 1). Those highly pure endothelial cells (~90%) expressed endothelial markers PECAM1 and CDH5 (Figs. 4A–B). The endothelial identity was further verified by the formation of capillary-like structures on Matrigel matrix *in vitro* (Fig. 4C). When the endothelial cells were dissociated on day 5 and injected into immunodeficient mice, we were able to detect robust formation of vessel-like structures in the injected plugs (Fig. 4D; H&E stain). The human origin and the transplantation potential of the differentiated endothelial cells were proven by staining with an antibody specific for human PECAM1 (Fig. 4D; IHC stain), which demonstrated their successful integrating into host vessels and forming arborizing vascular patterns.

## Discussion

Two major objectives during endothelial differentiation are to make the system free of stress and animal substances. To this end, we used ES medium minus the active ingredients as the basal medium because its identical nutrient/salt compositions to ES medium minimized the stress arising upon differentiation. To free the media from animal proteins, recombinant insulin from yeast, transferrin from rice, and recombinant VEGF/Activin A/bFGF from *E. coli* were chosen. Further, recombinant vitronectin from *E. coli* was used for coating. The replacement minimized the carryover of growth factors that could inhibit the endothelial differentiation^22^. Further, bovine serum was routinely used for endothelial differentiation because its repertoire of growth factors maintains cell survival and helps with the differentiation^46^. We excluded serum by providing all required growth factors for the cell survival/endothelial differentiation, and by substituting albumin with a synthetic anti-adsorptive. The exclusion of serum avoids issues of batch-to-batch variation and allows for more refined endothelium-driving pathways.

Besides the advantages in large-scale production and application, the rapid formation of human endothelial cells means more than technical advance. For a specific type of somatic cell to form during embryogenesis, a series of cellular crosstalk involving several intermediate lineages is required. Stepwise mutual inductions between cell lineages explain the longer time required to form endothelial cells with the previous methods. The rapid formation of endothelial cells with our method suggests some of the intermediate cell types were bypassed during endothelial differentiation, which in turn suggests we are approaching to understanding the endothelial differentiation in a paracrine-free, cell-autonomous manner.

The fewer required exogenous factors in the completely defined system helped clarify the intracellular events for pluripotent cell-to-mesoderm transition. The formation of the endothelial precursor required only Activin A, a TGFβ agonist, and CHIR99021, an activator of canonical Wnt pathway. Although the activation of canonical WNT pathway alone triggered the expression of T, the induced cells failed to express other mesodermal markers such as HAND1 and KDR, and were unable to be differentiated into endothelial cells. Because Activin A was required at this step, we suspect SMAD2 or SMAD3, 2 downstream mediators of TGFβ signaling, interacted with β-catenin or T for mesodermal transition^47^. By pulling down and sequencing the targets of SMADs, β-catenin, and T, we are able to understand how the 2 pathways converge to trigger the gene regulator network of mesoderm. It is also interesting to see if *in vitro* gastrulation still occurs at a single cell-density with our minimum-noise culture system to further validate the sufficiency of the 2 signaling pathways.

Only a fraction of cells on day 3 were KDR+ hemangioblasts, which indicates incomplete iPS cell-to-mesoderm conversion or partial hemangioblast formation in mesoderm. If some iPS cells remained undifferentiated even under the identical induction, it would be interesting to see if they were either fixed in pluripotency or just not yet in a permissive window to form mesoderm. The 2 possibilities will be distinguished by isolating and re-differentiating the refractory population. However, it is more likely that hemangioblasts accounted for only a part of the induced mesoderm. One possibility for the mixed output is that the differentiation was asynchronous and the early differentiated cells affected the fates of the later. The other possibility is that the formation of hemangioblasts was stochastic and dependent on the variation of certain genes inside cells.

VEGF is a dominant trigger of endothelial fate due to its ability to sustain ERK activation^48^. However, the necessity of FGF at a low density indicates paracrine FGF signaling mediates endothelial formation through a distinct yet critical manner. At a low density, the colonies formed with VEGF alone, albeit very few in number, were not significantly smaller in size compared with those with VEGF plus bFGF. The discrepancy between the number and the size of colonies suggests FGF mediated the survival or attachment of hemangioblasts before endothelial fate was determined. How FGF, with similar intercellular mediators to VEGF, plays such a distinct role is worth exploring. FGF might be required to prime the sufficient expression of KDR in the mesoderm/hemangioblasts^49^, or a unique intracellular mediator of FGF might be required for the survival of hemangioblasts. For the former, it is interesting to identify how FGF triggers KDR expression intracellularly. For the latter, identifying the unique survival pathway downstream of FGF will pave a way for long-term maintenance of endothelial precursors in the future.

Although BMP signaling was found essential for the formation of murine endothelium in a serum-free system^26^, exogenous BMP was not required in our human system. A simple yet unlikely explanation is that the requirement for BMP is species-specific to the murine system. BMP4 has been shown to boost endothelial differentiation from human ES cells^50^ and was one of the critical mediators in a chemically defined mesodermal induction medium for the formation of human endothelial precursors^44^. If BMP signaling was the essential driver for both species, sufficient auto- or paracrine BMP activity selectively occurred in the human system may explain the discrepancy. It is also possible that BMP signaling was not required for both species. The basal medium for murine differentiation contained several animal-derived substances, and among them the concentration of albumin was so high (~1.25 mg/ml)^51^ that it might contain inhibitory factors for endothelial differentiation. The inhibitory signals could be antagonized by BMP4^52^. Besides, only half of VEGF concentration was used in the murine system. It is possible that BMP4 supplemented the insufficient VEGF activity in driving endothelial formation. In this case, we suspect the ERK downstream of BMP4 might serve the role^25^,^53^.

The endothelial-enrichment effect could be served by PVA, PF68, and BSA, all of which have an anti-adsorption function. The significant reduction in the number of adherent cells with them in culture suggested the anti-adsorptives inhibited the attachment of cells. The enrichment resulted within 6 hours after replating, which indicates hemangioblasts were the target of selection. The presence of non-hemangioblast lineages with both VTN and PVA indicates PVA did not exert its enrichment by killing undesirable cell types. More likely, hemangioblasts, rather than other cell types, selectively attached to the surface coated with anti-adsorptives and the rest of the cells just died through anoikis. The similar purity achieved with molecules of different structures and molecular compositions suggests they did not result from a particular receptor. Possibly, hemangioblasts might engulf the anti-adsorptives on the plastic surface to access and attach to the bottom. This hypothesis could be tested by measuring the intake of dye-conjugated anti-adsorptives by hemangioblasts versus that by the other cell types in culture.

Overall, we identified a minimal set of signals to acquire high-purity human endothelial cells from iPS and ES cells in a system that is simple, rapid, and animal substance-free. The generality of the method was validated by testing on multiple pluripotent cell lines across different racial origins. The functionality of the differentiated endothelial cells was also demonstrated with classical *in vitro* and *in vivo* assays. Together with a chemically defined and non-integrating system to obtain iPS cells from somatic cells, our scalable method offers unlimited supplies of isogenic endothelial cells for clinical applications. Further, the intermediate cells gained during differentiation could also serve as a source for other cell lineages with proper modifications in drivers^54^. Since the system is totally defined, in need of only few factors, and compatible with low-density differentiation, it is also well suited for deciphering the intracellular events and regulatory networks that determine the endothelial fate.

## Methods

### List of Materials

Human iPS cell line DF19-9-7T (WiCell)^8^; human ES cell line TW1 (Bioresource Collection and Research Center, Taiwan); human ES cell line Ch8 (National Engineering Research Center of Human Stem Cells, China); Activin A (Prospecbio); CHIR-99021 and Y-27632 (SelleckChem); mouse VEGF-A (Prospecbio); human VEGF-A (Prospecbio); VEGF-E (Prospecbio); VEGF-B (Prospecbio); basic FGF (ACROBiosystems); insulin from *Saccharomyces cerevisiae* (Sigma); transferrin from Oryza sativa (Invitria); anti-human PECAM1 (eBioscience); anti-human KDR and anti-human PDGFRA (BD Bioscience); Matrigel matrix (BD Bioscience); poly(vinyl alcohol) (Sigma 360627); Pluronic F-68 (Sigma); bovine albumin fraction V (Invitrogen).

### Culture of human ES cells and iPS cells

The culture of ES/iPS cells essentially followed the protocol^10^. Briefly, ES/iPS cells were seeded at 30,000 cell per well in a 12-well plate precoated with 1250 ng/cm^2^ of vitronectin (VTN). The ES medium, TeSR-E8, was replaced daily. For the first 24 hours after seeding, 10 µM of Y-27632 was included to enhance survival^55^. The cells were split upon at a subconfluent density (~1,000,000 per well in a 12-well plate). Only cell lines of less than 60 passages were used for studies.

### Differentiation of ES/iPS cells into endothelium

The basal medium for differentiation was composed of 12 g/L of DMEM/F-12, 3.56 g/L of HEPES, 1.742 g/L of sodium bicarbonate, 14 ug/L of sodium selenite, 10.7 mg/L of recombinant transferrin, 19.4 mg/L of recombinant insulin, and 64 mg/L of L-Ascorbic acid 2-phosphate sesquimagnesium salt hydrate. For mesoderm induction, iPS/ES cells were seeded in a mesoderm inducer (MI: basal medium with 10 µM of Y-27632, 250 ng/cm^2^ of VTN, 3 µM of CHIR99021, and 2 ng/ml of Activin A). For mesoderm-to-endothelium transition, the induced mesoderm was dissociated and seeded in vaculogenic mixture (VM: basal medium with 2 mg/ml of PVA plus 20 ng/ml of mVEGF-A in figures 2 and 3 or 10 ng/ml of hVEGF-A in figure 4) for additional 72 hours. The endothelial cells were harvested on day 5. The factors and their concentrations used to optimize MI and VM are presented in respective figure legends. Unless otherwise specified, all differentiations were conducted in the wells of 12-well plates; 20,000 iPS cells and 30,000 mesodermal cells were used for mesodermal transition and endothelial differentiation, respectively. The concentration of various factors used: VTN: 250 ng/cm^2^, precoated; bovine serum albumin (BSA): 350 µg/ml; Pluronic F-68 (PF68): 2 mg/ml; PVA: 2 mg/ml; mVEGF-A: 20 ng/ml; hVEGF-A: 10 ng/ml; VEGF-B: 20 ng/ml; VEGF-E: 100 ng/ml; basic FGF: 20 ng/ml.

### Flow cytometry and cell sorting

For PECAM1 and CDH5 staining, the cells were dissociated with TrypLE on day 5. The dissociated cells were spun down and resuspended in ice-cold flow buffer (PBS+0.1% BSA). Small aliquots of cells were used for counting and the rest were used for staining and flow cytometry by the recommended concentrations and procedures. For PDGFRA or KDR flow cytometry, 0.5 ul of the PE-conjugated anti-human PDGFRA or KDR antibody were inoculated into the culture 48 hours (Fig. 1E) or 72 hours (Figs. 2D–E) after the initiation of differentiation. After incubated with the antibodies for 3 hours, the cells were dissociated, pelleted, and resuspended in flow buffer for sorting with BDAria FACS sorter (Fig. 1E) or direct assay by flow cytometry (Figs 2D–E). FL1 channel was included in Fig. 1E to let the cell populations more distinguishable.

### Immunofluorescence

The cells were fixed on day 5 with 1% paraformaldehyde in PBS for 5 min. After washed twice with PBS, they were incubated in blocking buffer (PBS+0.5% BSA+0.1% Tween-20) for 20 min. The blocking buffer was replaced with blocking buffer plus 5 µg/ml of anti-human PECAM1 antibody and incubated for 30 min. After washed 3 times with blocking buffer, the cells were incubated with blocking buffer plus 0.5 µg/ml of horseradish peroxidase-conjugated anti-mouse antibody for 30 min. After washed twice with blocking buffer and once with tyramide buffer (PBS+0.1% Tween-20+1 mM imidazole), the cells were incubated in tyramide buffer plus 2 µM of rhodamine-tyramide and 0.003% of hydrogen peroxidase for 10 min^56^. After washed twice with blocking buffer, the cells were incubated in PBS plus 1 µg/ml of Hoechst 33258 for image documentation.

### Matrigel capillary assay

The iPS/ES cells (40,000) were induced with MI per well of a 6-well plate for 48 hours. After dissociation, they (60,000) were induced with VM per well of **a** 6-well plate for additional 72 hours. On day 5, the cells were dissociated with Accutase and pelleted. The pellets were resuspended and 45,000 cells were seeded per well of **a** 96-well plate. Each well contained basal medium plus 20 ng/ml of bFGF and 60 µL of solidified Matrigel. Image documentation was done 16 hours later after fixation with 4% paraformaldehyde in PBS.

### Matrigel plug assay

The iPS/ES cells (300,000) were induced with MI in **a** 10-cm dish for 48 hours. After dissociation, they (900,000) were induced with VM in a 10-cm dish for additional 72 hours. On day 5, the cells were dissociated with Accutase and pelleted. The cells (500,000 for TW1, 1,000,000 for Ch8 and DF19-9-7T) were resuspended in 25 ul of basal medium plus 200 ng of bFGF and 5 µg of heparin sulfate and mixed with 100 µL of ice-cold Matrigel matrix for injection subcutaneously in mice (NOD.CB17-*Prkdc^scid^*/J). The plugs were harvested 7 days later, fixed in 4% formaldehyde in PBS and embedded in paraffin after dehydration. The use of animals was approved by the Animal Care and Use Committee at National Cheng Kung University.

### Immunohistochemistry for PECAM1 on Matrigel plugs

Immunohistochemistry (IHC) was performed on 4-μm-thick formalin-fixed paraffin-embedded sections. Monoclonal mouse anti-human CD31 antibody (1:100 dilution, Genemed) was used as the primary antibody. The procedures were done with the Bond-Max Automated IHC stainer (Leica Biosystems Newcastle Ltd, Australia) according to the following protocol. Tissues were deparaffinized with xylene and pre-treated with the Epitope Retrieval Solution 2 (EDTA buffer, pH 9.0) at 100°C for 20 min, followed by mouse on mouse blocking buffer(Leica) incubated at room temperature for 30 min. Then, the primary antibody was incubated at room temperature for 30 min. Subsequently, tissues were incubated with post primary at room temperature for 8 min and polymer for 8 min and hydroperoxide blocking for 5 min before they were developed with 3,3′- diaminobenzidine chromogen for 10 min using the Bond Polymer Refine Detection Kit (Leica Biosystems Newcastle Ltd, United Kingdom). Counterstaining was carried out with hematoxylin.

### RT-qPCR

The mRNA was isolated by adding 100 ul of the lysis buffer without DNase I (Single Cell-to-CT kit, Invitrogen 4458237) directly to the sorted cells or the adherent cells on dish. After incubating at 37C for 20 min, the digestion was stopped by adding stop buffer. Fifteen µL of the lysates were used as the templates for reverse transcription with MMLV reverse transcriptase (Enzymatics) and Oligo d(T)23VN. The cDNA was used for SYBR-based RT-qPCR assays (supplementary Table 1). The fluorescence data was analyzed by “qpcR” package with the following parameters: baseline = 1:8, norm = TRUE, methods = “sigfit”, model = l5, type = “Cy0”, which.eff = “sig”, type.eff = “mean.pair”, which.cp = “Cy0”^57^. The means and standard deviations from permutation analysis were exported for further statistical analysis.

### Statistical analysis

Student's *t*-test was used to compare 2 groups. One-way ANOVA with Tukey's post-hoc test was used for multiple groups. P values of less than 0.05 were considered statistically significant (*P<0.05, **P<0.01, ***P<0.001).

## Author Contributions

Y.W. and I.Y. collected data; Y.W. and C.S. prepared all figures; Y.W., K.T., and P.C. analyzed and interpreted data; S.H. provided study materials; I.S. and P.C. conceived and designed experiments; P.C. wrote the main manuscript text. All authors reviewed the manuscript.

## Supplementary Material

Supplementary Information Supplementary Information

## Figures and Tables

**Table 1:** Quantification of endothelial formation with three independent ES/iPS cell lines.

**Cell line:**		DF19-9-7T	Ch8	TW1
**Day 2**	**Number of cells:**	24444 ± 1951	19629 ± 1336	25183 ± 2089
**Day 5**	**Number of cells:**	18889 ± 1001	23888 ± 1211	37777 ± 2819
	**Numbers of Endothelial cells**: (Percentage of Endothelial cells)	17614 ± 462 (93.5 ± 2.9%)	22860 ± 1302 (95.6 ± 0.6%)	21770 ± 8961 (88.2 ± 1.2%)

Per well of a 12-well plate, iPS (DF19-9-7T) and ES (Ch8, and TW1) cells (10,000) were induced with MI for 48 hours and replated (30,000) in VM for additional 72 hours. The cells were harvested on day 2 for counting and day 5 for counting and flow cytometry with a PE-conjugated anti-PECAM1 antibody. (Data are mean ± s.e.m., n = 3)
