# Supplementary material for: Defining Minimum Essential Factors to Derive Highly Pure Human Endothelial Cells from iPS/ES Cells in an Animal Substance-Free System
Source: Sci Rep. 2015 Apr 13;5:9718. doi: 10.1038/srep09718 (PMC4394195; doi:10.1038/srep09718)
Supplement: Supplementary Information [file srep09718-s1.pdf]

Defining Minimum Essential Factors to Derive Highly Pure Human Endothelial Cells from iPS/ES Cells in an Animal Substance-Free System

Yu-Ting Wu, I-Shing Yu, Kuen-Jer Tsai, Chien-Yu Shih, Shiaw-Min Hwang, Ih-Jen Su, Po-Min Chiang

	Forward Primer	Reverse Primer
NANOG	TCGTATTTGCTGCATCGTAA	CTCATTGAAACACTCGGTGA
CDH1	GATTTTTCGGCAGTTCAAGC	TTTCCACCCCCAAAGAAAAT
CLDN3	GACCGCCAATACTTGACC	AAATATCAAGTGCCCCTTCC
CDH2	GAAAGGAAAGAAAGGGGTGG	TGTCAGAAGTCTCTCCAGTT
VIM	AAGAAACAGCTTTCAAGTGC	TGTAGGAGTGTCGGTTGTTA
ITGB1	TACGCGAGTCTTACTTTGAG	ACTAGTGTGAAACAAGATGGG
FN1	GCTAGTTTACCGTTCAGAAGTA	TTCAATGAAGGAAAGGTGGAG
ZEB1	TATTCCTTCCCCACTAGGAAC	ATCTGTTGTCCCAACTTATGC
SNAI2	GATGGGAATAAGTGCAAAAGAG	CACTTGGAAGGGGTATTGTAT
TWIST1	AAGGCATCACTATGGACTTTC	TAACTGACTATGGTTTTGCAGG
PDGFRA	GCACATTAACTGTTGCACTT	CACACCATTGTTTTGGGAAC
T	GATGCAGTGACTTTTTGTCG	CTTCTTAACCTGAGACTGCC
HAND1	CTCAGGTGTTTGTTCATCCT	CAAGAGCATAGACACTGCTT
KDR	CCCATCCTCAAAGAAGTAGC	AACACTTACATTGCCTGGTT
GATA4	GAAGTCTTTTGTCCAGGAGG	TTAGCAGTCGTCTTCTTTCC
GATA2	TCCCGAGCTTAGATTCTGTA	GGTCACTACATCAGCACAAT
PDGFRB	TACCCCAAGAAGGATGTGAG	GGGCAGTGACAAAACCATA
ETV2	GAATTACGAGAAGCTGAGCC	GAAGCGGTACGTGTACTTTC
FLI1	AGGGTAACACTAAGTACCTTCT	CTTTTTAAATGGCAGGGTGC
ACTA2	AACAGGAATACGATGAAGCC	GGCATAATTCCACAGGACAT
TAL1	GTGATTTCGATGGTACGTGA	AGTACAACGTTGACGGAAAG
CDH5	ATCAGGAGTGACAGATCACA	GAGTCTCAAAGCAAGGTCTC
TEK	TTTATCCCTCACCTGTAGCA	AGATGTTAGCATCCTGGACT
LMO2	TTTCAATCACTGTCCTCCAC	TCTCCATGCAGTTTTCCTTG
PECAM1	GCCCTAGAAGCCAATTAGTC	TACCTTTCAGAGACCTCCTC
GAPDH	ACAAGAGGAAGAGAGAGACC	CCTCTTCAAGGGGTCTACAT

Supplemental Table 1. Primers used for RT-qPCR.
